# Oxidative Stress and Its Impact on Reperfused Myocardium: Pathophysiological Insights and Therapeutic Perspectives

**DOI:** 10.3390/cells15131185

**Published:** 2026-06-29

**Authors:** Iris Bararu Bojan, Carmen Plesoianu, Maria-Cristina Vladeanu, Stefan Dobreanu, Dragos-Florin Tesoi, Codruta Badescu, Cezar Ilie Foia, Otilia Elena Frasinariu, Dan Iliescu, Oana Viola Badulescu, Codruta Olimpiada Iliescu Halitchi, Amin Bazyani, Manuela Ciocoiu

**Affiliations:** 1 Department of Morpho Functional Sciences, Faculty of Medicine, Grigore T. Popa University of Medicine and Pharmacy, 16 Universitatii Street, 700115 Iasi, Romaniadragos-florin.tesoi@email.umfiasi.ro (D.-F.T.); manuela.ciocoiu@umfiasi.ro (M.C.); 2 Department of Internal Medicine II, Faculty of Medicine, Grigore T. Popa University of Medicine and Pharmacy, 16 Universitatii Street, 700115 Iasi, Romania; 3 Department of Surgical Sciences, Faculty of Medicine, Grigore T. Popa University of Medicine and Pharmacy, 16 Universitatii Street, 700115 Iasi, Romania; 4 Department of Pediatrics, Faculty of Medicine, Grigore T. Popa University of Medicine and Pharmacy, 16 Universitatii Street, 700115 Iasi, Romania; 5 Heart Institute Prof. Dr. George I.M. Georgescu Iasi, 50 Bd. Carol I, 700503 Iasi, Romania

**Keywords:** myocardial ischemia–reperfusion injury, oxidative stress, reactive oxygen species

## Abstract

Myocardial ischemia–reperfusion injury (MIRI) represents a major contributor to morbidity and mortality in patients undergoing reperfusion therapy after acute myocardial infarction. Although timely restoration of coronary blood flow is essential for myocardial salvage, reperfusion paradoxically initiates a complex cascade of molecular and cellular events that may aggravate myocardial injury. Oxidative stress is considered one of the central mechanisms underlying MIRI, primarily through excessive production of reactive oxygen species (ROS) and reactive nitrogen species (RNS), leading to mitochondrial dysfunction, calcium overload, endothelial injury, inflammatory activation, and cardiomyocyte death. This review summarizes the current understanding of the pathophysiological mechanisms involved in oxidative stress-mediated reperfusion injury, with emphasis on mitochondrial permeability transition pore opening, inflammasome activation, cytokine release, neutrophil extracellular trap formation, macrophage polarization, and interconnected cell death pathways including PANoptosis. Emerging evidence regarding immunometabolic regulation and epigenetic modulation in MIRI is also discussed. In addition, current pharmacological and non-pharmacological cardioprotective strategies targeting oxidative stress, mitochondrial dysfunction, and inflammatory signaling are reviewed, highlighting both promising experimental findings and the persistent challenges in clinical translation. A deeper understanding of the molecular interplay between oxidative stress and inflammatory pathways may facilitate the development of integrated therapeutic approaches aimed at improving myocardial recovery and long-term cardiovascular outcomes following reperfusion therapy.

## 1. Introduction

Reperfusion therapy remains the cornerstone strategy for preserving ischemic myocardium following coronary occlusion. Nevertheless, the restoration of blood flow may paradoxically contribute to additional myocardial damage, a phenomenon known as reperfusion injury. During reperfusion, the sudden reintroduction of oxygen promotes excessive oxidative stress, intracellular calcium accumulation, and metabolic disturbances related to tissue acidosis, all of which contribute to cardiomyocyte dysfunction and death.

Cardiomyocytes are particularly susceptible to oxidative injury because of their high mitochondrial content and intense respiratory activity, which represent major sources of reactive oxygen species (ROS). Excessive ROS generation can disrupt cellular membrane stability, leading to myocardial enzyme release and structural cellular damage. In addition, oxidative stress activates mitochondrial-dependent apoptotic pathways, thereby amplifying cardiomyocyte loss and promoting infarct expansion.

Several mechanisms have been implicated in ROS-mediated apoptosis, including alterations in mitochondrial permeability transition pore function and the activation of pro-apoptotic signaling pathways. Given that mitochondria occupy a substantial proportion of cardiomyocyte volume, mitochondrial dysfunction plays a central role in the pathophysiology of ischemia–reperfusion injury and subsequent impairment of cardiac function [1].

Early restoration of coronary blood flow through reperfusion strategies such as thrombolytic therapy or primary percutaneous coronary intervention (PPCI) remains the cornerstone of treatment for patients with ST-segment elevation myocardial infarction (STEMI). Rapid coronary recanalization is essential for limiting ischemic myocardial necrosis by restoring oxygen delivery and allowing recruitment of inflammatory and reparative cells necessary for tissue healing.

Despite its undeniable clinical benefits, reperfusion may paradoxically induce additional myocardial injury, a phenomenon known as myocardial ischemia–reperfusion injury (MIRI). This process has been recognized for several decades and involves multiple pathological mechanisms occurring within the first minutes after reperfusion. These include cardiomyocyte death through apoptosis, necrosis, necroptosis, and pyroptosis, disturbances in cellular energy metabolism associated with mitochondrial dysfunction, oxidative stress, intracellular calcium overload, amplified inflammatory responses, and endothelial injury leading to coronary microvascular dysfunction. Collectively, these events contribute to enlargement of the final infarct size and additional damage to residual viable myocardium.

A more comprehensive understanding of the temporal progression and molecular mechanisms underlying MIRI may facilitate the development of novel therapeutic strategies aimed at reducing myocardial damage and preventing adverse ventricular remodelling and heart failure. Reflecting this evolving perspective, a recent expert consensus document published by the Canadian Cardiovascular Society proposed a novel classification of atherothrombotic myocardial infarction treated with reperfusion therapy. This classification is based on the progressive structural and molecular alterations occurring within the myocardium during ischemia and reperfusion, with the objective of promoting the development of tissue injury-specific therapeutic approaches for myocardial infarction.

Interest in the immuno-inflammatory mechanisms associated with MIRI intensified following landmark clinical trials demonstrating that modulation of inflammatory signaling pathways can reduce recurrent cardiovascular events. Since then, research efforts have increasingly focused on oxidative stress, mitochondrial dysfunction, endothelial injury, and immune-mediated pathways as potential therapeutic targets. Current investigations aim to identify pharmacological and non-pharmacological interventions capable of complementing reperfusion therapy and providing synergistic cardioprotective effects [2,3].

## 2. Material and Methods

This narrative review was conducted to evaluate the role of oxidative stress in reperfused myocardium and its contribution to ischemia–reperfusion injury. A comprehensive literature search was performed using the electronic databases PubMed, Scopus, Web of Science, and Google Scholar. Relevant articles published in English up to March 2026 were considered for inclusion. This review builds upon the authors’ ongoing research interests in cardiovascular pathophysiology, oxidative stress, inflammation, and coronary artery disease. Through previous studies investigating the pathophysiology of atherosclerotic disease, and cardiovascular risk factors, the authors have developed a particular interest in the mechanisms linking ischemic injury, inflammation, and adverse cardiac remodelling, which provided the rationale for the present review.

The search strategy included combinations of the following keywords and Medical Subject Headings (MeSH) terms: “oxidative stress”, “ischemia-reperfusion injury”, “reperfused myocardium”, “myocardial reperfusion”, “reactive oxygen species”, “cardiomyocyte apoptosis”, “mitochondrial dysfunction”, “mitochondrial permeability transition pore”, “myocardial infarction”, and “reperfusion therapy”. Boolean operators (“AND”, “OR”) were used to optimize the search process.

Original research articles, experimental studies, clinical studies, systematic reviews, and meta-analyses investigating oxidative stress mechanisms during myocardial reperfusion were included. Particular attention was given to studies evaluating reactive oxygen species generation, mitochondrial alterations, inflammatory responses, calcium overload, apoptotic pathways, and antioxidant therapeutic strategies. Reference lists of selected articles were additionally screened to identify further relevant publications.

Articles not directly related to myocardial ischemia–reperfusion injury, studies lacking adequate scientific relevance, conference abstracts without full text availability, and non-English publications were excluded.

The retrieved studies were analyzed qualitatively, with emphasis placed on the molecular mechanisms underlying oxidative stress-induced myocardial injury, the role of mitochondrial dysfunction, and emerging therapeutic approaches aimed at reducing reperfusion-associated cardiac damage.

## 3. Clinical Determinants and Phenotypes of MIRI in Contemporary STEMI Care

Myocardial ischemia–reperfusion injury (MIRI) contributes significantly to morbidity and mortality following successful reperfusion therapy in STEMI. Clinically, reperfusion injury manifests through several interrelated phenotypes, including myocardial stunning, microvascular obstruction (no-reflow), reperfusion arrhythmias, intramyocardial hemorrhage, and acute hemodynamic deterioration.

Myocardial stunning is characterized by transient but reversible post-ischemic ventricular dysfunction despite restoration of coronary blood flow. It results primarily from calcium overload, oxidative stress, and impaired excitation–contraction coupling, leading to temporary reductions in systolic and diastolic performance.

The no-reflow phenomenon reflects inadequate myocardial perfusion despite successful reopening of the infarct-related artery. Its pathogenesis involves endothelial injury, distal embolization, inflammatory cell infiltration, tissue edema, and microvascular obstruction. No-reflow is associated with larger infarct size, adverse ventricular remodeling, and worse clinical outcomes.

Reperfusion arrhythmias commonly occur during the first hours after revascularization and include accelerated idioventricular rhythm, ventricular tachyarrhythmias, atrial fibrillation, and sinus node disturbances. These rhythm abnormalities are largely driven by abrupt ionic shifts, calcium overload, oxidative stress, and autonomic activation.

Lethal reperfusion injury represents irreversible cardiomyocyte death triggered by reperfusion itself, mainly through mitochondrial dysfunction and opening of the mitochondrial permeability transition pore. Experimental studies suggest that this mechanism may account for a substantial proportion of the final infarct size.

Intramyocardial hemorrhage (IMH), detected primarily by cardiac magnetic resonance imaging, represents a severe form of reperfusion-related microvascular damage. Frequently coexisting with microvascular obstruction, IMH is associated with larger infarcts, reduced left ventricular function, adverse remodeling, and an increased risk of major adverse cardiovascular events.

Finally, reperfusion injury may present with hemodynamic instability, including hypotension, bradyarrhythmias, cardiogenic shock, and persistent slow-flow or no-reflow. Early recognition of these manifestations is crucial, as they identify patients at increased risk of heart failure, ventricular remodeling, and unfavorable long-term outcomes [4,5].

## 4. Ischemic Time Dependency

The severity of myocardial ischemia–reperfusion injury (MIRI) is strongly dependent on the duration of ischemia before reperfusion. Although timely restoration of coronary blood flow remains the cornerstone of STEMI management, prolonged ischemic times increase the susceptibility of cardiomyocytes and the coronary microcirculation to reperfusion-mediated damage. Experimental and clinical studies have demonstrated that delayed reperfusion is associated with greater oxidative stress, mitochondrial dysfunction, calcium overload, and inflammatory activation, ultimately resulting in larger infarct size and reduced myocardial salvage. Consequently, symptom-to-balloon time remains one of the most important determinants of both infarct size and the extent of reperfusion injury [6,7].

## 5. Anterior STEMI: A High-Risk MIRI Phenotype

Anterior STEMI, typically resulting from proximal left anterior descending artery occlusion, represents the phenotype most vulnerable to severe reperfusion injury. The large myocardial territory at risk, combined with higher oxygen demand and extensive microvascular involvement, predisposes these patients to larger infarcts, greater microvascular obstruction, and increased incidence of intramyocardial hemorrhage. Cardiac magnetic resonance (CMR) studies consistently demonstrate that anterior infarctions exhibit a greater burden of MIRI-related complications and are more frequently associated with adverse ventricular remodelling and subsequent heart failure [4,8].

## 6. Distal Embolization and Microvascular Obstruction

Microvascular obstruction (MVO) is a frequent complication of STEMI despite successful epicardial reperfusion, with reported incidences ranging from approximately 25% to 56%, depending on the diagnostic modality used, including cardiac magnetic resonance imaging (MRI), angiography, and echocardiography. Similarly, angiographic no-reflow has been observed in approximately one-quarter of patients undergoing primary PCI.

Microembolization of thrombotic and atherosclerotic debris during PCI is considered a major contributor to MVO. Early studies demonstrated an association between elevated intracoronary microparticle levels and the presence of MVO, suggesting a potential role for circulating microparticles in microvascular injury. However, these findings have not been consistently replicated and their clinical significance remains uncertain.

Increasing evidence supports the involvement of platelet activation in the development of MVO. Higher platelet reactivity, increased ADP-induced platelet aggregation, and enhanced platelet–leukocyte interactions have all been associated with a greater incidence of MVO. Activated platelets may contribute to microvascular dysfunction through distal microthrombus formation and the release of vasoconstrictive mediators, particularly thromboxane A_2_. Consistent with this mechanism, antithrombotic therapies such as aspirin and heparin have been associated with lower rates of no-reflow and reduced microvascular injury in STEMI patients undergoing PCI [9].

Collectively, these findings suggest that microembolization, platelet activation, and microthrombus formation play important roles in the pathogenesis of MVO.

Distal embolization of thrombotic and atherosclerotic debris during primary percutaneous coronary intervention contributes significantly to microvascular dysfunction. Embolized particles obstruct downstream arterioles and capillaries, triggering endothelial injury, inflammatory cell recruitment, vasoconstriction, and tissue edema. These mechanisms culminate in microvascular obstruction (MVO), one of the hallmark manifestations of reperfusion injury. Importantly, MVO may occur despite successful restoration of epicardial coronary flow and is recognized as an independent predictor of larger infarct size, impaired left ventricular recovery, and adverse cardiovascular outcomes [10].

## 7. Coronary Physiology: IMR and CFR

The invasive assessment of coronary microvascular function has become increasingly important in characterizing reperfusion injury. The index of microcirculatory resistance (IMR) provides a quantitative and reproducible measure of microvascular resistance independent of epicardial coronary stenosis. Elevated IMR values after primary PCI correlate strongly with CMR-defined microvascular obstruction, larger infarct size, and reduced left ventricular function. Coronary flow reserve (CFR), which reflects the capacity of the coronary circulation to augment blood flow during hyperemia, offers complementary information regarding both epicardial and microvascular integrity. Together, IMR and CFR allow early identification of patients with severe microvascular dysfunction who may be at increased risk for adverse remodeling and heart failure [11].

## 8. Cardiac Magnetic Resonance Markers of MIRI

Cardiac magnetic resonance imaging is currently considered the reference standard for the non-invasive assessment of reperfusion injury. Beyond quantifying infarct size and myocardial salvage, CMR can identify several characteristic markers of MIRI, including microvascular obstruction, intramyocardial hemorrhage, myocardial edema, and area at risk. Among these, MVO and intramyocardial hemorrhage are the strongest predictors of adverse prognosis. Patients demonstrating these findings exhibit lower left ventricular ejection fractions, greater ventricular dilatation, and increased rates of major adverse cardiovascular events during follow-up [12].

## 9. MIRI and Heart Failure Development

Increasing evidence suggests that MIRI plays a central role in the transition from acute myocardial infarction to chronic heart failure. Through a combination of cardiomyocyte death, persistent microvascular dysfunction, inflammation, and adverse extracellular matrix remodeling, reperfusion injury contributes to progressive ventricular dilatation and systolic dysfunction. Patients with extensive MVO, elevated IMR values, or CMR evidence of intramyocardial hemorrhage are particularly prone to adverse left ventricular remodeling. Consequently, MIRI is increasingly recognized not only as an acute complication of STEMI but also as a major determinant of long-term heart failure risk and cardiovascular mortality [13].

## 10. Myocardial Ischemia Reperfusion Injury and Oxidative Stress: Mitochondrial Dysfunction and Calcium Overload

The restoration of oxygen supply during reperfusion following myocardial ischemia leads to a sudden and marked increase in the production of reactive oxygen species (ROS) and reactive nitrogen species (RNS). Excessive accumulation of these oxidant molecules overwhelms endogenous antioxidant defenses and ROS scavenging systems, resulting in oxidative stress, cellular injury, and activation of inflammatory pathways.

Reduction of oxygen generates the superoxide anion (O^2−^), which can subsequently give rise to highly reactive hydroxyl radicals (OH) through reactions involving hydrogen peroxide (H_2_O_2_) or via the Fenton reaction. Reactive nitrogen species generated during MIRI include nitric oxide (NO), nitrogen dioxide (NO_2_), and peroxynitrite (ONOO^−^). Several enzymatic and cellular systems contribute to ROS production during reperfusion injury, including NADPH oxidase, xanthine oxidase, nitric oxide synthase (NOS), and mitochondria.

Among these sources, mitochondria represent one of the most important contributors to oxidative stress during MIRI. A key mechanism involves mitochondrial succinate accumulation during ischemia, followed by rapid oxidation upon reperfusion, which activates reverse electron transport (RET) at Complex I of the mitochondrial respiratory chain. This process results in substantial production of superoxide anions. Complex I releases O^2−^ into the mitochondrial matrix, whereas Complex III contributes to ROS release into the intermembrane space.

NADPH oxidase also plays a major role in myocardial oxidative stress. This enzyme complex consists of membrane-bound NOX catalytic subunits associated with cytosolic regulatory proteins. NOX2 serves as the principal catalytic component in phagocytic NADPH oxidase. Following activation, cytosolic subunits translocate toward the membrane-bound gp91phox protein, assembling an active oxidase complex capable of generating superoxide radicals. Importantly, NADPH oxidase expression has also been demonstrated in cardiomyocytes, where it contributes significantly to redox imbalance during reperfusion injury [14,15].

Excessive ROS production activates multiple intracellular signaling pathways associated with inflammation and cell death, including apoptosis and pyroptosis. Experimental studies demonstrated that ROS generation may directly activate the nucleotide-binding oligomerization domain-like receptor protein 3 (NLRP3) inflammasome pathway, thereby amplifying inflammatory responses and promoting pyroptotic cell death.

Oxidative stress is also one of the major triggers of mitochondrial dysfunction during MIRI. In particular, ROS promote opening of the mitochondrial permeability transition pore (mPTP), a critical event in reperfusion injury. Opening of the mPTP disrupts mitochondrial membrane integrity and facilitates the release of Cytochrome C (CytC) into the cytosol, thereby initiating apoptotic signaling cascades. Schriewer et al. demonstrated that ROS are essential for mPTP opening and further showed that mPTP activation itself may amplify ROS production, creating a vicious cycle of oxidative damage. Their study additionally identified poly (ADP-ribose) polymerase-1 (PARP) as an important mediator of mitochondria-dependent necrosis during ischemia/reperfusion injury. PARP appears to contribute to oxidative stress amplification and may facilitate mPTP opening through poly ADP-ribosylation of mitochondrial targets. Conversely, mPTP opening may further enhance PARP activation, although the precise sequence of these events remains incompletely understood. Based on these findings, Schriewer et al. proposed PARP inhibition as a potential therapeutic strategy capable of attenuating reperfusion injury even after mPTP opening has occurred.

Another major consequence of mPTP opening is intracellular calcium overload. Excessive accumulation of Ca^2+^ ions is a hallmark of reperfusion injury and contributes substantially to cardiomyocyte dysfunction and death. Elevated cytosolic calcium concentrations activate multiple apoptotic pathways and further aggravate mitochondrial damage.

**Figure 1:**
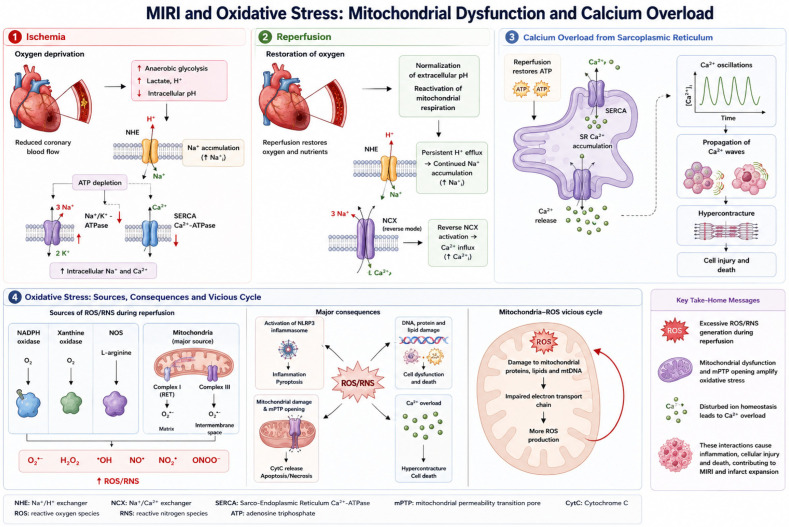
MIRI and Oxidative Stress: Mitochondrial Dysfunction and Calcium Overload. Reperfusion triggers excessive generation of reactive oxygen and nitrogen species (ROS/RNS) from mitochondria, NADPH oxidase, and other cellular sources, overwhelming endogenous antioxidant defenses. Oxidative stress promotes mitochondrial permeability transition pore (mPTP) opening, calcium overload, inflammasome activation, and apoptotic signaling, creating a self-amplifying cycle of mitochondrial dysfunction, inflammation, and cardiomyocyte death.

During the ischemic phase, impaired aerobic metabolism and suppression of the Krebs cycle force myocardial cells to switch toward anaerobic glycolysis, leading to lactate accumulation and intracellular acidosis due to increased H^+^ concentrations. Under these acidic conditions, activation of the Na^+^/H^+^ exchanger (NHE) promotes extrusion of H^+^ ions in exchange for Na^+^ influx, resulting in intracellular sodium accumulation. Simultaneously, ischemia-induced ATP depletion inhibits the activity of Na^+^/K^+^-ATPase and Sarco-Endoplasmic Reticulum Calcium ATPase (SERCA Ca^2+^-ATPase), further contributing to sodium overload and impaired calcium homeostasis. These ionic disturbances ultimately favor intracellular Ca^2+^ accumulation, thereby exacerbating oxidative stress, mitochondrial dysfunction, and cardiomyocyte injury during reperfusion—Figure 1 [2,16,17].

During reperfusion, restoration of oxygen availability reactivates mitochondrial oxidative metabolism and contributes to normalization of extracellular pH. Under these conditions, the Na^+^/H^+^ exchanger (NHE) remains active, continuously extruding H^+^ ions from the cardiomyocytes while promoting persistent intracellular Na^+^ accumulation. Elevated intracellular sodium concentrations subsequently activate the reverse mode of the Na^+^/Ca^2+^ exchanger (NCX), resulting in excessive intracellular Ca^2+^ influx and calcium overload.

Additional disturbances in calcium homeostasis involve the sarcoplasmic reticulum (SR). Recovery of ATP production during reperfusion restores SR activity and facilitates cyclic uptake and release of Ca^2+^ ions. This process generates intracellular calcium oscillations, and the released Ca^2+^ may propagate between adjacent SR structures, leading to calcium wave formation. Excessive calcium wave propagation contributes to hypercontracture of cardiomyocytes, a phenomenon strongly associated with irreversible cellular injury and cell death.

The abrupt increase in ROS generation caused by renewed oxygen delivery during reperfusion therefore represents one of the major mechanisms disrupting cellular homeostasis. These highly reactive molecules interfere with multiple intracellular pathways and organelles, inducing widespread cellular dysfunction. Mitochondria are particularly vulnerable to oxidative injury, and mitochondrial damage further amplifies ROS production, establishing a self-perpetuating cycle of oxidative stress and cardiomyocyte injury during MIRI [18,19].

## 11. Molecular Mechanisms of Oxidative Stress and Epigenetic Regulation in Myocardial Ischemia Reperfusion Injury

Myocardial ischemia remains one of the leading causes of cardiovascular morbidity and mortality worldwide. Although reperfusion therapies have substantially improved survival by restoring coronary blood flow, the process of reperfusion itself may paradoxically aggravate myocardial damage, a phenomenon known as myocardial ischemia–reperfusion injury (MIRI). During ischemia, reduced coronary perfusion deprives cardiomyocytes of oxygen and metabolic substrates, leading to impaired ATP production, ionic imbalance, and cellular dysfunction. Once blood flow is re-established, the abrupt restoration of oxygen triggers a cascade of detrimental biological responses that further compromise myocardial integrity.

MIRI contributes significantly to adverse ventricular remodelling, impaired cardiac contractility, and the progression toward heart failure, thereby exerting a major impact on long-term prognosis. Despite considerable advances in cardiovascular medicine, effective therapies specifically targeting reperfusion injury remain limited. The pathophysiology of MIRI is multifactorial and involves interconnected mechanisms such as oxidative stress, intracellular calcium overload, mitochondrial dysfunction, inflammatory activation, and metabolic disturbances. However, the complex molecular interactions underlying these processes are not yet fully elucidated, emphasizing the need for further mechanistic investigations and novel therapeutic targets [20].

Among emerging regulatory pathways, RNA epigenetic modifications have attracted growing attention. N6-methyladenosine (m6A) represents the most abundant internal RNA modification in eukaryotic cells and plays a central role in post-transcriptional gene regulation. This modification is dynamically controlled by methyltransferases (“writers”), demethylases (“erasers”), and RNA-binding proteins (“readers”), which together modulate RNA stability, translation efficiency, degradation, and alternative splicing. Increasing evidence indicates that m6A methylation participates in the regulation of oxidative stress responses, inflammatory signaling, mitochondrial homeostasis, and cardiomyocyte survival during MIRI [21,22].

A key regulator within this pathway is the fat mass and obesity-associated protein (FTO), an RNA demethylase capable of removing m6A modifications from target transcripts. Through this activity, FTO influences multiple cellular processes by altering RNA fate and protein expression. Recent studies have demonstrated that FTO expression is markedly reduced under ischemia–reperfusion conditions both in vivo and in vitro. Experimental findings suggest that FTO exerts cardioprotective effects by stabilizing survival-related transcripts through m6A demethylation. One proposed mechanism involves the demethylation of yes-associated protein 1 (YAP1) mRNA, which enhances transcript stability and promotes cardiomyocyte resistance to ischemic injury.

Furthermore, increased FTO expression has been associated with attenuation of ischemia-induced m6A accumulation and preservation of cardiac contractile function. FTO-mediated demethylation appears to protect critical contractile protein transcripts from degradation during ischemic stress, thereby maintaining myocardial function. Additional evidence suggests that FTO may also suppress pyroptosis during ischemia–reperfusion injury by modulating the stability of Cbl Proto-Oncogene (CBL) mRNA and preventing β-catenin degradation through inhibition of ubiquitin-mediated pathways. Despite these observations, the precise mechanisms through which FTO regulates oxidative stress and mitochondrial integrity in reperfused myocardium remain incompletely understood.

Mitochondrial dysfunction represents a pivotal component of reperfusion injury, and peroxisome proliferator-activated receptor gamma coactivator-1 alpha (PGC-1α) has emerged as a major regulator of mitochondrial homeostasis. PGC-1α functions as a transcriptional coactivator involved in mitochondrial biogenesis, energy metabolism, antioxidant defense, and regulation of reactive oxygen species production. Reduced expression of PGC-1α has been associated with enhanced oxidative damage and worsening metabolic dysfunction [22,23].

PGC-1α also interacts with nuclear respiratory factor 2 (Nrf2) to stimulate mitochondrial transcription factor A (TFAM), thereby supporting mitochondrial DNA replication and transcription. Experimental studies have shown that activation of PGC-1α can alleviate oxidative stress, apoptosis, and inflammation during MIRI while preserving mitochondrial dynamics and function. Pharmacological activation of PGC-1α has demonstrated beneficial effects by restoring redox balance, reducing tissue remodeling, and limiting cardiomyocyte death.

**Figure 2:**
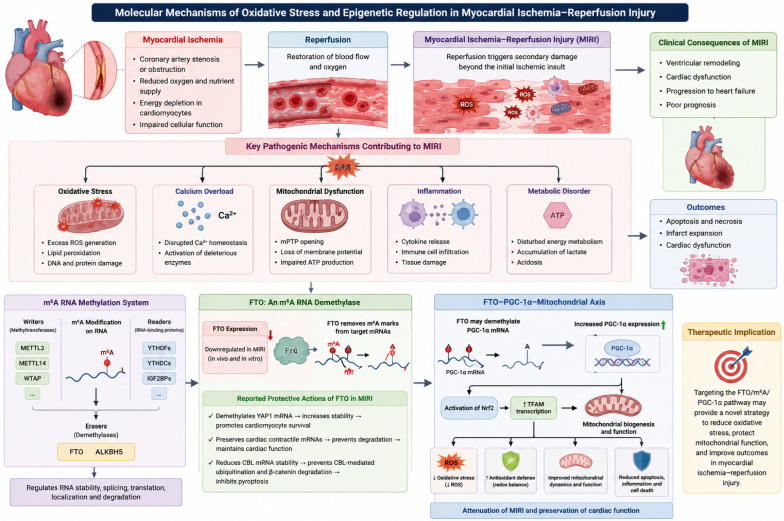
Molecular Mechanisms of Oxidative Stress and Epigenetic Regulation in Myocardial Ischemia–Reperfusion Injury. Ischemia–reperfusion induces alterations in m6A RNA methylation and downregulation of the RNA demethylase FTO, contributing to oxidative stress, inflammatory activation, pyroptosis, and cardiomyocyte injury. FTO-mediated stabilization of protective transcripts, including YAP1, may enhance cellular survival and preserve myocardial function. In parallel, PGC-1α acts as a central regulator of mitochondrial biogenesis, antioxidant defenses, and energy metabolism through interactions with Nrf2 and TFAM. The proposed crosstalk between FTO-dependent epigenetic regulation and PGC-1α-mediated mitochondrial protection may represent a novel cardioprotective mechanism and a potential therapeutic target in MIRI.

Given these findings, increasing attention has been directed toward the potential interaction between FTO-mediated m6A regulation and PGC-1α signaling pathways. Further research is needed to determine whether FTO directly modulates mitochondrial function and oxidative stress in reperfused cardiomyocytes through epigenetic regulation of PGC-1α and related mitochondrial protective mechanisms [24]—Figure 2.

**Key Takeaway:** FTO and PGC-1α represent promising regulators of cellular adaptation to ischemia–reperfusion stress through epigenetic and mitochondrial protective mechanisms. However, evidence is currently limited to preclinical studies, and their clinical relevance in STEMI patients remains to be established.

## 12. Neutrophil Heterogeneity and NET-Mediated Inflammation in MIRI

Neutrophils are among the earliest immune cells recruited to the myocardium following myocardial ischemia–reperfusion injury (MIRI), playing a central role in the initiation and amplification of the acute inflammatory response. After reperfusion, these cells rapidly migrate into the injured cardiac tissue, where they release large amounts of pro-inflammatory cytokines, reactive oxygen species (ROS), and proteolytic enzymes that contribute to cardiomyocyte injury and tissue remodeling.

A major advancement in neutrophil biology has been the discovery of neutrophil extracellular traps (NETs), specialized extracellular web-like structures composed of decondensed chromatin, histones, and granule-derived proteins. NETs are generated in response to inflammatory and ischemic stimuli and have emerged as important mediators of myocardial damage during MIRI. Through their cytotoxic components, NETs directly injure cardiac tissues while simultaneously promoting platelet aggregation, thrombus formation, and microvascular obstruction. In addition, the inflammatory mediators embedded within NETs intensify local inflammation and enhance coagulation pathways, thereby aggravating myocardial injury. Experimental evidence has further demonstrated that deficiencies in cardioprotective pathways, including ALDH2 and histidine decarboxylase signaling, may exacerbate MIRI by enhancing NET-associated inflammatory mechanisms [25].

Neutrophils exhibit remarkable phenotypic and functional diversity as a consequence of their differentiation status, maturation stage, and environmental activation signals. This heterogeneity explains their dual and sometimes contradictory roles in myocardial injury and tissue repair. While activated neutrophils can amplify inflammation and oxidative damage, they may also contribute to tissue healing by promoting angiogenesis and reparative responses. Consequently, broad therapeutic strategies aimed at suppressing neutrophil activity have shown limited efficacy because they may simultaneously inhibit beneficial reparative functions.

The dynamic plasticity of neutrophils and the redundancy of inflammatory signaling pathways highlight the importance of identifying specific pathogenic neutrophil subsets involved in detrimental post-ischemic responses, particularly those associated with NET formation. Recent advances in single-cell RNA sequencing (scRNA-seq) have revealed the existence of distinct neutrophil subpopulations with specialized inflammatory and immunomodulatory functions. Studies in murine myocardial infarction models identified novel neutrophil clusters, including SiglecF-positive and YM1High neutrophils, which appear to participate in regulating inflammatory and anti-inflammatory responses within the injured myocardium. However, the characterization of pro-inflammatory neutrophil populations in human MIRI remains insufficiently explored [26].

To address these gaps, recent investigations analyzed neutrophils isolated from patients with myocardial infarction following percutaneous coronary intervention and compared them with cells obtained from healthy individuals using single-cell transcriptomic approaches. These studies identified a distinct MMP9High neutrophil subset strongly associated with NET formation, enhanced inflammatory activity, and increased cardiac injury. Furthermore, SPI1 was identified as a critical transcription factor regulating CST7 expression and promoting the differentiation of these pathogenic neutrophils. These findings emphasize the complexity of neutrophil heterogeneity in MIRI and suggest that targeting specific NET-forming neutrophil subsets may represent a promising therapeutic strategy for reducing inflammation-mediated myocardial damage [27].

Despite these promising findings, the therapeutic relevance of NET modulation in MIRI should be interpreted cautiously. Current evidence supporting NET involvement is mainly derived from experimental models and mechanistic animal studies, where NET inhibition, DNase administration, PAD4 blockade, or modulation of upstream inflammatory pathways has been associated with reduced myocardial inflammation, smaller infarct size, and improved microvascular perfusion. In humans, however, the evidence remains largely indirect and biomarker-based, relying on circulating markers such as cell-free DNA, citrullinated histone H3, myeloperoxidase–DNA complexes, and neutrophil elastase activity, which have been associated with infarct severity, thrombotic burden, microvascular obstruction, and adverse outcomes after myocardial infarction.

Importantly, validated interventional data in patients with STEMI are still lacking. No NET-targeted therapy has yet demonstrated consistent clinical benefit in randomized human trials focused on MIRI prevention or post-infarction remodeling. Moreover, NET formation represents only one component of a complex inflammatory network involving platelets, complement activation, endothelial dysfunction, monocyte recruitment, oxidative stress, and coagulation. Therefore, although NET-forming neutrophil subsets represent an attractive therapeutic target, their clinical translation is limited by the absence of standardized NET biomarkers, uncertainty regarding optimal timing of intervention, potential impairment of host defense, and incomplete characterization of pathogenic neutrophil phenotypes in human MIRI.

Thus, NET-targeted strategies should currently be regarded as an experimental and hypothesis-generating approach rather than an established therapeutic option. Future translational studies should clearly distinguish mechanistic evidence from animal models, observational human biomarker associations, and true interventional efficacy before NET inhibition can be incorporated into clinical cardioprotective strategies.

## 13. Emerging Evidence on MMP9High Neutrophils in MIRI

Recent studies investigating neutrophil heterogeneity in MIRI have identified distinct pro-inflammatory neutrophil subsets that appear to play a major role in myocardial injury progression. Among these populations, MMP9High neutrophils have attracted increasing attention due to their strong association with inflammatory activation and tissue damage. Transcriptomic analyses demonstrated that these cells possess a molecular profile markedly different from neutrophils with low MMP9 expression, suggesting the existence of specialized neutrophil phenotypes during ischemia–reperfusion injury.

Current evidence indicates that IFIT1High neutrophils may represent an intermediate or less inflammatory phenotype capable of differentiating into MMP9High neutrophils under ischemic and inflammatory conditions. Validation studies performed in patients with MIRI confirmed a significant increase in circulating MMP9High neutrophils, accompanied by a reduction in IFIT1High neutrophils. Furthermore, neutrophils from patients with MIRI exhibited enhanced MMP9 expression and reduced IFIT1 expression, supporting the concept of a phenotypic shift toward a more pro-inflammatory state during reperfusion injury.

Importantly, the expansion of MMP9High neutrophils has been closely associated with the severity of myocardial damage. Several investigations reported positive correlations between the proportion of these neutrophils and established biomarkers of cardiac injury, including cardiac troponin T and lactate dehydrogenase. In addition, elevated levels of MMP9High neutrophils were linked to increased systemic inflammatory markers such as white blood cell count, neutrophilia, and neutrophil-to-lymphocyte ratio, further emphasizing their involvement in the inflammatory cascade characteristic of MIRI.

Experimental and clinical studies also demonstrated increased MMP9 expression at both RNA and protein levels in neutrophils isolated from patients with MIRI. Circulating serum MMP9 concentrations were similarly elevated and positively associated with the abundance of MMP9High neutrophils, suggesting that these cells may serve as a continuous source of pro-inflammatory mediators capable of amplifying myocardial inflammation and tissue injury.

**Figure 3:**
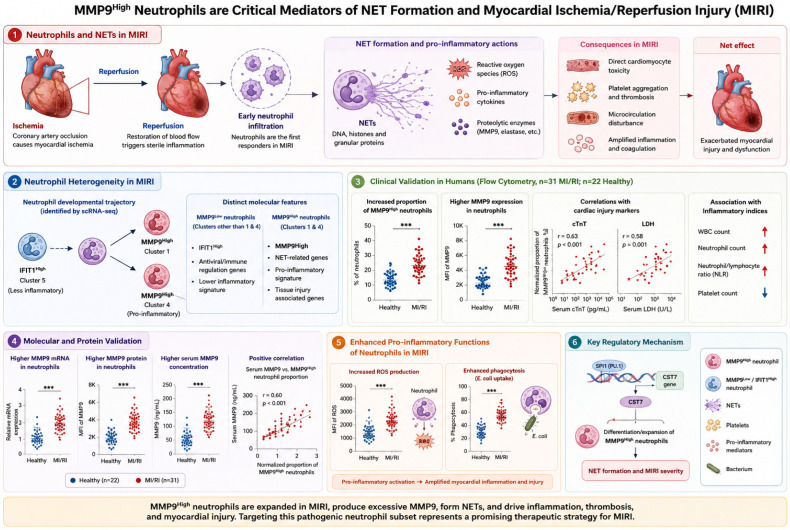
MMP9High Neutrophils and NET-Mediated Inflammation in Myocardial Ischemia–Reperfusion Injury (MIRI): Mechanisms, Clinical Correlations, and Therapeutic Implications. Ischemia–reperfusion promotes the phenotypic transition of neutrophils toward a pro-inflammatory MMP9High subset characterized by enhanced matrix metalloproteinase-9 expression, increased reactive oxygen species production, and augmented inflammatory activity. Expansion of MMP9High neutrophils is associated with elevated cardiac injury biomarkers, systemic inflammation, endothelial dysfunction, and microvascular damage. These findings highlight neutrophil heterogeneity as a key determinant of post-ischemic inflammation and identify MMP9High neutrophils as a potential therapeutic target in MIRI. *** *p* ≤ 0.001.

Beyond their molecular profile, MMP9High neutrophils display enhanced inflammatory functionality. These cells exhibit increased production of reactive oxygen species and augmented phagocytic activity, features that may contribute to oxidative stress, endothelial dysfunction, and microvascular injury within the reperfused myocardium. Collectively, these findings highlight the critical role of neutrophil heterogeneity in MIRI and suggest that selective targeting of pathogenic neutrophil subsets, particularly MMP9High neutrophils, may represent a promising therapeutic strategy for limiting inflammation-driven cardiac injury [25,28]—Figure 3.

## 14. Macrophage Polarization, Metabolic Reprogramming, and HSP90-Related Signaling in MIRI

Macrophages represent key regulators of the inflammatory response and tissue repair processes during myocardial ischemia–reperfusion injury (MIRI). In the acute phase of injury, classically activated M1 macrophages predominate and contribute to the amplification of inflammation through the release of pro-inflammatory cytokines and cytotoxic mediators, thereby aggravating myocardial damage. As the reparative phase progresses, alternatively activated M2 macrophages become increasingly dominant, promoting inflammation resolution, tissue remodeling, angiogenesis, and infarct healing through the expression of anti-inflammatory and reparative genes [29].

Recent evidence has highlighted the close relationship between macrophage metabolism, polarization state, and functional activity in the injured myocardium. Metabolic reprogramming of macrophages has emerged as a critical determinant of their inflammatory phenotype and their interactions with cardiomyocytes and fibroblasts during post-ischemic remodeling. Consequently, strategies aimed at shifting macrophage polarization from the pro-inflammatory M1 phenotype toward the reparative M2 phenotype are increasingly viewed as promising therapeutic approaches in MIRI.

Several metabolic intermediates have recently been implicated in macrophage-mediated cardioprotection. Malic acid (MA), an intermediate of the tricarboxylic acid–glyoxylic acid cycle involved in cellular energy metabolism, has been associated with modulation of inflammatory responses and cardioprotective effects during ischemia–reperfusion injury. Experimental studies demonstrated that MA-containing conditioned media, as well as MA itself, may attenuate oxygen–glucose deprivation/reoxygenation-induced injury in cardiomyocytes, suggesting a protective role against ischemic stress. However, the precise molecular targets and mechanisms responsible for these effects remain incompletely elucidated [30].

Another important metabolite involved in macrophage metabolic reprogramming is itaconic acid (ITA), which is generated from cis-aconitate in the Krebs cycle through increased expression of aconitate decarboxylase 1 (ACOD1). ITA has gained considerable attention because of its potent anti-inflammatory properties observed in several inflammatory disease models. Multiple molecular targets have been proposed for ITA, including NLRP3 inflammasome signaling, the Keap1–Nrf2 antioxidant pathway, glyceraldehyde-3-phosphate dehydrogenase (GAPDH), and Janus kinase 1 (JAK1). In the context of MIRI, elevated ITA production has been associated with attenuation of myocardial injury and suppression of inflammatory signaling [31].

Recent investigations have identified pyruvate kinase M2 (PKM2) as an important downstream target involved in the cardioprotective effects of ITA. PKM2 is a key glycolytic enzyme responsible for ATP generation and is strongly linked to inflammatory metabolic pathways. Enhanced PKM2-dependent glycolysis has been associated with increased production of inflammatory mediators and activation of inflammasome pathways in macrophages. Beyond its metabolic role, PKM2 also exerts non-glycolytic functions related to cell survival and transcriptional regulation. Experimental data suggest that mitochondrial translocation of PKM2 may promote interaction with the anti-apoptotic protein Bcl2, thereby reducing cardiomyocyte apoptosis under ischemia–reoxygenation conditions.

Glycogen synthase kinase 3 beta (GSK3β), a constitutively active serine/threonine kinase involved in apoptosis, differentiation, and inflammatory regulation, has also emerged as an important mediator of macrophage polarization in MIRI. Inhibition of GSK3β activity appears to suppress M1-associated inflammatory markers while enhancing M2 macrophage polarization. Recent studies demonstrated that pharmacological agents such as PBC may inhibit GSK3β through increased phosphorylation at the Ser9 residue, thereby favoring anti-inflammatory macrophage reprogramming and enhancing expression of ACOD1 and MDH2, enzymes associated with ITA and MA production [32].

Heat shock protein 90 (HSP90), a molecular chaperone belonging to the stress-induced heat shock protein family, has also been implicated in cardioprotection during ischemia–reperfusion injury. HSP90 regulates multiple signaling pathways involved in apoptosis, inflammation, and oxidative stress. Experimental evidence indicates that HSP90 may suppress activation of caspase-dependent apoptotic pathways, modulate complement-related inflammatory signaling, and influence macrophage polarization through interactions with p38 and GSK3β signaling cascades. Interestingly, recent findings suggest that PBC may exert cardioprotective effects not by directly targeting GSK3β, but rather by enhancing the interaction between HSP90 and GSK3β, ultimately leading to inhibition of GSK3β activity and promotion of M2 macrophage polarization. Silencing HSP90 attenuated these beneficial effects, further supporting the central role of HSP90-mediated signaling in macrophage metabolic regulation and cardioprotection during MIRI.

**Figure 4:**
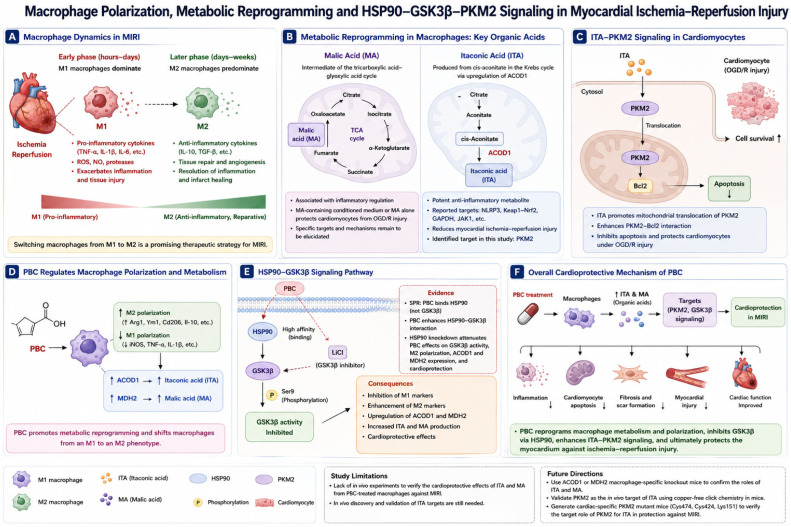
Macrophage Polarization, Metabolic Reprogramming, and HSP90–GSK3β–PKM2 Signaling Pathways in Myocardial Ischemia–Reperfusion Injury.

Collectively, these findings emphasize the growing importance of macrophage metabolic reprogramming, mitochondrial signaling, and HSP90-associated pathways in the pathogenesis of MIRI. Targeting macrophage phenotype transitions and associated metabolic networks may therefore represent a promising strategy for reducing inflammation, limiting cardiomyocyte apoptosis, and improving myocardial recovery following reperfusion injury [33]—Figure 4.

**Key Takeaway:** Macrophages play a dual role in MIRI, with pro-inflammatory M1 macrophages predominating during the acute injury phase and reparative M2 macrophages contributing to tissue healing and remodeling. Emerging evidence indicates that macrophage metabolism is a major determinant of this phenotypic switch. Experimental studies have identified several metabolic regulators—including itaconic acid (ITA), malic acid (MA), PKM2, GSK3β, and HSP90—as modulators of macrophage polarization and inflammatory activity. These pathways may reduce myocardial injury by promoting anti-inflammatory macrophage reprogramming, suppressing inflammasome activation, and limiting cardiomyocyte apoptosis. However, current evidence is derived almost exclusively from preclinical studies, and the clinical relevance of targeting macrophage metabolic rewiring in STEMI patients remains to be established. Future translational studies are needed to determine whether modulation of macrophage phenotype can improve myocardial recovery and long-term outcomes following reperfusion therapy.

## 15. Mitochondrial-Targeted Cardioprotective Therapies

Given the central role of mitochondrial dysfunction in the pathogenesis of myocardial ischemia–reperfusion injury, several therapeutic strategies have been developed to directly target mitochondrial integrity, oxidative stress, and bioenergetic preservation. Among these, the mitochondria-targeting peptide elamipretide (SS-31) has received considerable attention. Elamipretide selectively interacts with cardiolipin within the inner mitochondrial membrane, stabilizing mitochondrial structure, improving electron transport chain efficiency, reducing reactive oxygen species production, and preserving ATP generation. Experimental studies demonstrated reductions in infarct size and improved cardiac function following ischemia–reperfusion; however, clinical studies have yielded mixed results, and definitive evidence supporting routine clinical use remains lacking [34].

Another promising approach involves MitoQ, a mitochondria-targeted antioxidant consisting of ubiquinone linked to a lipophilic triphenylphosphonium cation that facilitates mitochondrial accumulation. By selectively scavenging mitochondrial reactive oxygen species, MitoQ has shown cardioprotective effects in experimental models, including attenuation of oxidative stress, preservation of mitochondrial function, and reduction in cardiomyocyte death. Nevertheless, clinical evidence in STEMI patients remains limited.

Additional mitochondrial-targeted peptides, including SS-31-derived compounds, continue to be investigated for their ability to preserve mitochondrial bioenergetics, prevent mitochondrial permeability transition pore opening, and reduce oxidative injury during reperfusion. These agents represent an emerging class of therapies designed to directly address one of the fundamental mechanisms underlying MIRI [35].

More recently, mitochondrial transplantation has emerged as an innovative experimental strategy. This approach involves the delivery of viable exogenous mitochondria into ischemic myocardium with the aim of restoring cellular bioenergetics and limiting reperfusion-associated injury. Preclinical studies have reported improvements in ATP production, myocardial viability, and ventricular function following mitochondrial transplantation. Early clinical experiences in selected cardiac surgery populations have also suggested feasibility and safety. However, important challenges remain regarding mitochondrial sourcing, delivery methods, cellular uptake, long-term efficacy, and scalability before this strategy can be considered for routine clinical application.

**Key Takeaway:** Mitochondrial-targeted therapies represent one of the most biologically rational approaches to cardioprotection because they directly address mitochondrial dysfunction, a central mechanism of MIRI. While agents such as elamipretide (SS-31) and MitoQ have demonstrated encouraging preclinical results, clinical translation remains incomplete, and mitochondrial transplantation should currently be regarded as a highly experimental but potentially transformative therapeutic concept [36].

## 16. MIRI, NLRP3 Inflammasome, and Cytokine-Mediated Inflammatory Dysregulation

Inflammasomes are intracellular multiprotein complexes that play a fundamental role in regulating innate immune and inflammatory responses. These structures are activated after recognition of danger-associated molecular patterns (DAMPs) or pathogen-associated molecular patterns (PAMPs), which are generated during tissue injury or infection. Among the numerous inflammasome subtypes identified to date, the nucleotide-binding oligomerization domain (NOD)-like receptor protein 3 (NLRP3) inflammasome is the most extensively investigated in myocardial ischemia–reperfusion injury (MIRI) and is considered one of the major mediators of post-ischemic inflammation.

The NLRP3 inflammasome consists of three principal components: the NLRP3 sensor protein, apoptosis-associated speck-like protein containing a caspase recruitment domain (ASC), and pro-caspase-1. Structurally, the NLRP3 protein contains a central nucleotide-binding NATCH domain involved in oligomerization, a C-terminal leucine-rich repeat (LRR) region, and a pyrin domain (PYD). ASC similarly contains a PYD domain together with a caspase recruitment domain (CARD), facilitating interaction between NLRP3 and pro-caspase-1.

Activation of the NLRP3 inflammasome is closely linked to Nuclear Factor-kβ (NF-kβ) signaling. Following recognition of DAMPs or PAMPs by Toll-like receptor 4 (TLR4), NF-kβ becomes activated and induces transcription of NLRP3 as well as the precursor cytokines pro-IL-1β and pro-IL-18. Subsequently, activated caspase-1, generated through NLRP3-mediated cleavage of pro-caspase-1, converts these inactive precursors into mature IL-1β and IL-18. These cytokines strongly amplify inflammatory signaling by stimulating additional cytokine release, enhancing immune cell recruitment, and promoting extracellular matrix remodeling within the injured myocardium.

Experimental investigations have demonstrated marked activation of the NLRP3 inflammasome pathway during MIRI. Studies using hypoxia/reoxygenation models showed increased expression of NLRP3, ASC, caspase-1, IL-1β, IL-18, and TNF-α in cardiac fibroblasts following ischemic stress, further supporting the central contribution of inflammasome activation to myocardial inflammation.

Recent advances have revealed that regulation of the NLRP3 inflammasome is considerably more complex than previously recognized, involving multiple homeostatic and compensatory pathways. Consequently, preclinical studies investigating inflammasome modulation in MIRI have produced conflicting results. Kawaguchi et al. demonstrated in murine models that knockout mice lacking ASC or caspase-1 developed smaller infarct areas and exhibited reduced cytokine and interleukin expression compared with wild-type animals. Their work additionally confirmed the presence of ASC and caspase-1 within infarcted myocardial tissue, strongly implicating inflammasome activation in reperfusion-associated injury [37].

Additional studies subsequently confirmed that NLRP3 expression is markedly upregulated after myocardial infarction and that its deficiency may improve myocardial function and preserve coronary flow. Experimental observations also demonstrated expression of Toll-like receptors (TLRs) 1–4 and 9 by myocardial fibroblasts during ischemia–reperfusion injury. In ex vivo models, NLRP3 knockout animals showed preserved contractile function and smaller infarct sizes compared with wild-type controls.

Interestingly, later investigations from the same research group produced unexpected findings, demonstrating that knockout of NLRP3 or ASC could also result in larger infarct sizes following ischemia/reperfusion injury. These observations led to the hypothesis that the NLRP3 inflammasome may exert not only pro-inflammatory effects but also cardioprotective functions under certain conditions. One proposed mechanism involves interaction with the reperfusion injury salvage kinase (RISK) pathway, a major pro-survival signaling cascade composed primarily of phosphatidylinositol-3-OH kinase (PI3K)-Akt and extracellular regulated kinase (ERK)-1/2 signaling pathways [38].

Beyond inflammasome activation, Toll-like receptor signaling itself contributes substantially to the inflammatory response during MIRI. Activation of TLRs by DAMPs or PAMPs initiates multiple intracellular pathways capable of promoting inflammation, apoptosis, and myocardial dysfunction. TLR4 signaling has received particular attention because of its interaction with Myeloid differentiation protein 88 (MyD88), a central adaptor protein involved in downstream inflammatory signaling. Activation of the TLR4–MyD88 pathway promotes NF-kβ activation and stimulates release of inflammatory mediators including TNF-α, IL-6, and IL-1β [39].

Several experimental studies demonstrated that inhibition of MyD88 may attenuate reperfusion injury. Miao et al. reported that pharmacological suppression of MyD88 reduced inflammatory responses and infarct size in murine models of MIRI. Similarly, inhibition of MyD88 signaling has been associated with reduced severity of reperfusion injury following heart transplantation in experimental animal studies. MyD88 may also interact with additional Toll-like receptors, particularly TLR9, whose expression increases significantly during myocardial ischemia. Furthermore, release of mitochondrial DNA (mtDNA) secondary to severe oxidative stress may activate TLR-dependent inflammatory pathways and further stimulate NF-kβ signaling.

Taken together, these findings illustrate the remarkable complexity of the inflammatory response during MIRI. Multiple interconnected signaling pathways, cytokines, inflammasomes, and immune mediators interact simultaneously, often exerting both deleterious and protective effects depending on the cellular context and timing of activation. Although the NLRP3 inflammasome appears to represent one of the major drivers of inflammatory injury during reperfusion, evidence also suggests potential homeostatic and cardioprotective functions, complicating the development of targeted therapies. Further investigations are therefore required to better distinguish adaptive from maladaptive inflammatory responses and to identify novel molecular targets capable of limiting myocardial injury without interfering with essential reparative mechanisms—Table 1 [40].

Despite the substantial body of evidence implicating NLRP3 inflammasome activation in MIRI, the translational significance of these findings remains uncertain. Current knowledge is derived from several distinct levels of evidence that should be interpreted separately. Experimental studies conducted in isolated cardiomyocytes, cardiac fibroblasts, and hypoxia/reoxygenation models consistently demonstrate activation of the NLRP3–caspase-1–IL-1β/IL-18 signaling axis following ischemic stress. Animal studies further support a pathogenic role for inflammasome activation, as pharmacological inhibition or genetic deletion of NLRP3 pathway components has frequently been associated with reduced infarct size, attenuated inflammatory responses, and improved cardiac function. However, as discussed above, some experimental investigations have produced conflicting results, suggesting context-dependent cardioprotective functions of NLRP3 signaling and highlighting the complexity of inflammasome biology [41].

In humans, evidence remains predominantly observational. Elevated circulating concentrations of IL-1β, IL-18, caspase-1, and other inflammasome-related biomarkers have been associated with larger infarct size, impaired ventricular function, adverse remodeling, and worse clinical outcomes following myocardial infarction. Nevertheless, these associations do not establish causality and may reflect broader inflammatory activation rather than specific inflammasome-mediated injury.

Interventional evidence remains limited. Although anti-inflammatory strategies targeting the IL-1 signaling pathway have demonstrated cardiovascular benefits in selected patient populations, no therapy specifically targeting NLRP3 inflammasome activation has yet been validated for routine clinical use in STEMI patients undergoing reperfusion. Furthermore, most available clinical studies have focused on systemic inflammatory markers rather than direct assessment of myocardial inflammasome activity.

### Translational Limitations

Several factors currently limit the clinical translation of NLRP3-targeted therapies. First, the temporal dynamics of inflammasome activation during ischemia and reperfusion remain incompletely understood, and inhibition at different stages may produce divergent effects. Second, experimental models often fail to reproduce the complexity of human STEMI, including advanced age, diabetes, atherosclerosis, and concomitant pharmacological therapies. Third, emerging evidence suggests that NLRP3 signaling may participate not only in inflammatory injury but also in adaptive repair and tissue homeostasis, raising concerns that indiscriminate inhibition could impair beneficial healing responses. Finally, the absence of standardized biomarkers capable of accurately quantifying myocardial inflammasome activity in vivo complicates patient selection and therapeutic monitoring. Consequently, while the NLRP3 inflammasome remains a promising therapeutic target, current evidence supports its role primarily as a mechanistic and investigational pathway rather than an established clinical intervention for MIRI.

## 17. PANoptosis and Interconnected Cell Death Pathways in MIRI

Multiple forms of regulated cell death contribute to the pathogenesis of myocardial ischemia–reperfusion injury (MIRI), including pyroptosis, apoptosis, ferroptosis, and necroptosis. These pathways do not occur independently but rather interact through highly interconnected molecular networks that collectively amplify myocardial injury and inflammatory responses.

Recent advances in cell death biology introduced the concept of PANoptosis, a unique inflammatory cell death pathway characterized by the simultaneous activation and integration of pyroptotic, apoptotic, and necroptotic mechanisms. PANoptosis is mediated through a multiprotein complex capable of activating pyroptosis-associated caspases such as caspase-1, apoptosis-related caspases including caspase-8, as well as receptor-interacting serine/threonine kinase 3 (RIPK3) and mixed lineage kinase domain-like protein (MLKL), which are central mediators of necroptosis.

Unlike isolated forms of programmed cell death, PANoptosis is triggered by complex pathological stimuli such as ischemia, oxidative stress, and cytokine storms. Activation of this pathway results in extensive inflammatory amplification through the release of damage-associated molecular patterns (DAMPs) and pro-inflammatory cytokines, particularly IL-1β and IL-18. Consequently, PANoptosis represents a critical link between cell death and uncontrolled inflammatory activation during MIRI.

Experimental evidence suggests that simultaneous inhibition of multiple cell death pathways provides substantially greater cardioprotection compared with targeting individual pathways alone. Combined suppression of pyroptosis, apoptosis, and necroptosis has been associated with improved cell survival and reduced tissue injury, highlighting the therapeutic relevance of integrated cell death modulation.

**Figure 5:**
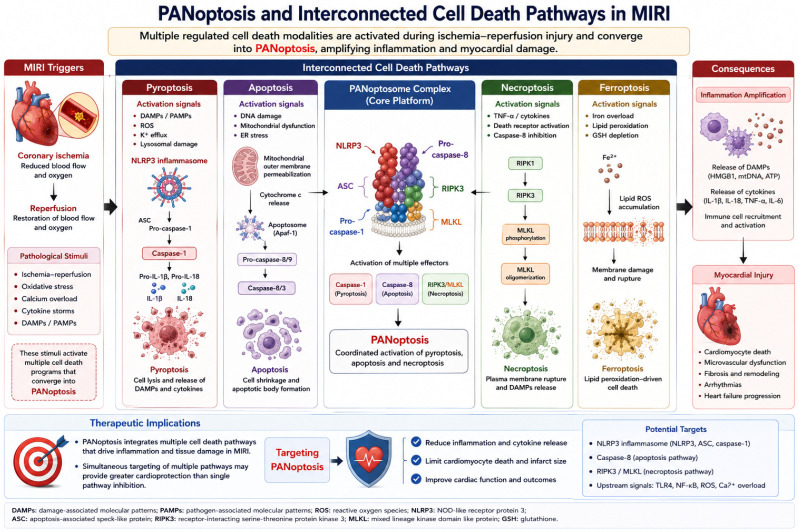
PANoptosis and Interconnected Cell Death Pathways.

Although research on PANoptosis in ischemia–reperfusion injury has thus far been more extensively explored in neurological disorders, growing evidence indicates that this pathway may also play an important role in cardiovascular diseases. Studies demonstrating beneficial effects of PANoptosis inhibition in doxorubicin-induced cardiotoxicity further support the possibility that targeting PANoptosis could represent a promising therapeutic strategy for limiting inflammation, reducing cardiomyocyte death, and attenuating myocardial damage during MIRI [42]—Figure 5.

Although PANoptosis has emerged as an attractive conceptual framework for understanding the interplay between multiple regulated cell death pathways, current evidence supporting its role in MIRI remains largely preliminary. Experimental studies have demonstrated extensive molecular crosstalk among pyroptotic, apoptotic, and necroptotic signaling pathways in cardiomyocytes exposed to ischemia/reperfusion conditions, providing a mechanistic basis for the existence of integrated cell death programs. Animal studies further suggest that simultaneous modulation of multiple cell death pathways may confer greater cardioprotection than inhibition of individual pathways alone, resulting in reduced infarct size, attenuated inflammation, and improved cardiac function [43].

However, direct evidence demonstrating the existence of PANoptosis as a distinct biological process in human myocardial ischemia–reperfusion injury is currently limited. Most available data originate from experimental models of cardiovascular injury, while human studies remain largely restricted to indirect observations of biomarkers associated with apoptosis, pyroptosis, necroptosis, and inflammatory activation. To date, no validated biomarkers specifically identify PANoptosis in patients with STEMI, and the relative contribution of integrated versus individual cell death pathways remains incompletely understood.

Interventional evidence is also lacking. While several experimental compounds targeting components of pyroptosis, apoptosis, or necroptosis have shown beneficial effects in preclinical models, no therapeutic strategy specifically designed to inhibit PANoptosis has demonstrated efficacy in clinical trials involving patients with acute myocardial infarction. Consequently, the potential therapeutic benefits of PANoptosis modulation remain hypothetical and require further validation [44].

### Translational Limitations

Several challenges currently hinder the translation of PANoptosis research into clinical practice. First, there is no universally accepted molecular definition or standardized method for identifying PANoptosis in vivo, making it difficult to distinguish this process from the concurrent activation of multiple independent cell death pathways. Second, most evidence originates from animal models or non-ischemic cardiovascular conditions, such as doxorubicin-induced cardiotoxicity, limiting direct extrapolation to human MIRI. Third, cell death pathways exhibit substantial temporal and spatial heterogeneity during myocardial infarction, and broad inhibition of these mechanisms may interfere with physiological processes involved in inflammation resolution and tissue repair. Therefore, while PANoptosis represents a promising conceptual model linking inflammation and cardiomyocyte death, its role in human MIRI should currently be regarded as investigational, and further mechanistic, translational, and clinical studies are required before PANoptosis-targeted therapies can be considered viable therapeutic strategies.

## 18. Ferroptosis

Ferroptosis is a distinct form of regulated cell death characterized by iron-dependent lipid peroxidation and excessive accumulation of reactive oxygen species (ROS), leading to irreversible cellular damage. Unlike apoptosis, ferroptosis is driven by disruption of redox homeostasis and can be inhibited by iron chelators and antioxidant compounds. Morphologically, ferroptotic cells exhibit characteristic mitochondrial abnormalities, including reduced mitochondrial volume, loss of cristae, increased membrane density, and membrane rupture.

A central regulator of ferroptosis is glutathione peroxidase-4 (GPX4), an antioxidant enzyme that detoxifies lipid peroxides and preserves membrane integrity. Depletion of glutathione (GSH), inhibition of the cystine/glutamate antiporter system (System Xc−), and impaired GPX4 activity promote lipid peroxide accumulation and ferroptotic cell death. Several interconnected pathways contribute to ferroptosis, including iron homeostasis, lipid metabolism, and mitochondrial oxidative stress.

Iron metabolism plays a crucial role in ferroptosis. Cellular iron uptake occurs through transferrin receptor-1 (TFR1) and divalent metal transporter-1 (DMT1), while ferritin serves as the primary intracellular iron storage complex. Disruption of iron homeostasis increases the labile iron pool, facilitating ROS generation through the Fenton reaction and promoting lipid peroxidation. Additional regulators, including heme oxygenase-1 (HO-1), iron-responsive element-binding proteins (IREBs), and heat shock protein B1 (HSPB1), further modulate intracellular iron availability and susceptibility to ferroptosis.

Recent evidence suggests that ferroptosis contributes significantly to myocardial ischemia–reperfusion injury by amplifying oxidative stress, mitochondrial dysfunction, inflammation, and cardiomyocyte death. Consequently, therapeutic strategies targeting iron overload, lipid peroxidation, and GPX4-dependent antioxidant defenses have emerged as promising approaches for limiting myocardial injury during reperfusion [45].

## 19. Clinical Modulation of MIRI: Current Therapeutic Strategies and Translational Challenges

A broad spectrum of therapeutic approaches, including both pharmacological and non-pharmacological interventions, is currently under investigation for the attenuation of myocardial ischemia–reperfusion injury (MIRI). Despite encouraging results obtained in experimental models, none of these strategies has yet become part of routine clinical practice. Most translational studies have focused on patients presenting with ST-segment elevation myocardial infarction (STEMI) undergoing primary percutaneous coronary intervention (PPCI), with investigational therapies administered immediately before or shortly after reperfusion.

The majority of clinical trials evaluated infarct size reduction as the principal endpoint, most commonly assessed by late gadolinium enhancement on cardiac magnetic resonance imaging. Additional outcomes frequently included serum biomarkers of inflammation, such as IL-1, IL-6, and TNF-α, markers of myocardial necrosis including troponin and CK-MB, as well as the incidence of major adverse cardiovascular events (MACEs). Nevertheless, many studies failed to demonstrate consistent clinical benefits. These disappointing translational results are likely multifactorial and may reflect difficulties in identifying the optimal therapeutic window, heterogeneity in clinical trial design, and overlap with standard cardioprotective therapies routinely administered before PPCI, particularly P2Y inhibitor antiplatelet therapy, which may obscure the isolated effects of the investigational intervention [46,47].

Although mechanistically diverse, most cardioprotective strategies against MIRI ultimately converge on several common pathophysiological targets. These include reduction in oxidative stress through limitation of reactive oxygen species (ROS) and reactive nitrogen species (RNS) generation, attenuation of inflammatory signaling, and preservation of mitochondrial integrity. Particular attention has been directed toward preventing mitochondrial permeability transition pore (mPTP) opening, stabilizing mitochondrial respiratory chain activity, and enhancing mitophagy, all of which are closely interconnected processes involved in reperfusion injury.

Among non-pharmacological interventions, myocardial conditioning strategies—including ischemic preconditioning, postconditioning, and remote ischemic conditioning—initially demonstrated substantial cardioprotective effects in experimental studies. Proposed mechanisms include modulation of nitric oxide synthase (NOS), reduction in ROS generation, and stabilization of intracellular pH. However, large randomized clinical trials evaluating ischemic postconditioning and remote ischemic conditioning failed to consistently reproduce these favorable outcomes in clinical settings. Nonetheless, some prospective studies and subsequent post hoc analyses suggested potential benefits of remote ischemic conditioning in selected patient populations, indicating that further refinement of these strategies may still be warranted.

Another emerging non-pharmacological approach involves intracoronary administration of hyperbaric supersaturated oxygen (SSO2) following PPCI. This strategy appears to improve endothelial function, increase oxygen delivery, stimulate endogenous antioxidant systems, and reduce cardiomyocyte apoptosis. Clinical studies demonstrated reductions in infarct size following SSO2 administration, supporting its potential cardioprotective role during reperfusion [31,32].

Therapeutic hypothermia has also attracted considerable interest because of its ability to reduce myofibrillar hypercontractility and preserve mitochondrial bioenergetics during reperfusion. Additional investigational approaches currently undergoing evaluation include extracorporeal cardiac shock wave therapy and other mechanical or device-based interventions designed to limit reperfusion-associated myocardial injury [48].

Pharmacological cardioprotection has similarly yielded heterogeneous results. Cyclosporine A, an mPTP-modulating agent, was investigated as a potential inhibitor of reperfusion injury but failed to demonstrate significant clinical efficacy in large trials. Likewise, sodium thiosulfate, which acts both as an antioxidant and hydrogen sulfide donor, did not significantly reduce infarct size or improve clinical outcomes in patients with STEMI [49].

Conversely, intravenous beta-blocker administration before reperfusion demonstrated more promising results. Studies evaluating early intravenous metoprolol administration before PPCI reported reductions in myocardial injury and improved cardiac outcomes. Proposed mechanisms include attenuation of matrix metalloproteinase activation and reduced degradation of sarcomeric proteins, thereby limiting contractile dysfunction associated with reperfusion injury. Similar observations obtained with Landiolol suggest that these cardioprotective effects may represent a broader class effect of beta-blockers [50].

More recently, Nicorandil, a hybrid ATP-sensitive potassium channel opener, demonstrated beneficial cardioprotective effects when administered intravenously before PCI in patients with STEMI. These findings further support the importance of mitochondrial and ion channel modulation in limiting reperfusion injury.

Other emerging therapeutic directions involve epigenetic and inflammatory modulation. Inhibition of Class-I histone deacetylases (HDACs), particularly with compounds such as Mocetinostat, has shown potential through stimulation of autophagy and enhancement of cellular survival pathways. Increasing attention has also been directed toward oxidized mitochondrial DNA (mtDNA), pyroptosis, and inflammasome-mediated injury. Reduction in oxidative stress may limit mtDNA damage and subsequent inflammatory activation, reinforcing the close interaction between oxidative injury and immune dysregulation during MIRI [51].

Consistent with these mechanisms, several studies targeted inflammatory mediators including NLRP3 inflammasome components, Toll-like receptor 9 (TLR9), and caspase-1 in an attempt to suppress IL-1β and IL-18 production and reduce pyroptosis. Among these agents, Tongxinluo, a traditional Chinese medicinal formulation, demonstrated promising experimental and clinical effects by attenuating endothelial pyroptosis, inhibiting caspase-1 activation, and reducing inflammatory cytokine release [52].

Several compounds already widely used in other clinical settings have also been evaluated for cardioprotective effects against MIRI. Metformin, despite negative results in some randomized trials involving non-diabetic STEMI patients, remains an attractive candidate because of its multiple beneficial mechanisms, including activation of AMP-activated protein kinase (AMPK), modulation of the reperfusion injury salvage kinase (RISK) pathway, attenuation of oxidative stress, reduction in apoptosis, and preservation of mitochondrial function.

Similarly, antioxidant therapies such as vitamin C and glutathione have shown encouraging effects in reducing oxidative stress and myocardial injury. High-dose intravenous vitamin C was associated with reduced cardiac enzyme release, improved endothelial function, and decreased ROS production in patients undergoing PCI or cardiac surgery. Glutathione administration before PCI also demonstrated potential benefits through scavenging of hydrogen peroxide and attenuation of oxidative myocardial damage [53,54].

Importantly, variability in therapeutic efficacy among clinical studies may also be influenced by metabolic comorbidities such as hypercholesterolemia and hyperglycemia, both of which appear capable of attenuating endogenous cardioprotective responses. Whether targeted metabolic modulation can specifically improve MIRI outcomes remains uncertain, particularly given the complex interactions between these metabolic disorders, atherothrombosis, inflammation, and reperfusion injury—Table 2.

## 20. Evidence Hierarchy and Translational Perspective of Cardioprotective Therapies

An important limitation of the current cardioprotection literature is the substantial heterogeneity in the level of evidence supporting individual therapeutic strategies. While many interventions have demonstrated efficacy in experimental models, only a small number have progressed to clinical evaluation, and even fewer have shown consistent benefits in large randomized trials. Consequently, therapies targeting MIRI should be interpreted according to their stage of translational development.

At the highest level of evidence are interventions that have undergone large clinical investigations. Among these, ischemic conditioning strategies and cyclosporine A initially generated considerable enthusiasm because of robust preclinical data and promising early clinical studies. However, subsequent large randomized trials failed to demonstrate consistent reductions in infarct size, heart failure, or mortality, highlighting the challenges of translating experimental cardioprotection into clinical practice.

A second category includes therapies supported by early clinical or phase II evidence but requiring further validation. Intravenous metoprolol administered before reperfusion, Nicorandil, hyperbaric supersaturated oxygen (SSO2), and selected antioxidant strategies have demonstrated encouraging effects on infarct size, myocardial salvage, or surrogate markers of reperfusion injury. Nevertheless, larger studies are needed to determine whether these benefits translate into improved long-term clinical outcomes.

Other approaches remain predominantly investigational. Metformin, despite its favorable mechanistic profile involving AMPK activation, mitochondrial protection, and modulation of RISK signaling, has produced mixed clinical results. Similarly, Tongxinluo has shown promising anti-inflammatory and anti-pyroptotic effects in experimental studies and selected clinical investigations, but broader international validation remains limited.

Finally, several strategies—including HDAC inhibition, NLRP3 inflammasome inhibition, TLR9 and caspase-1 targeting, mtDNA modulation, mitophagy enhancement, and other epigenetic or molecular interventions—remain largely confined to preclinical research. Although these approaches provide valuable mechanistic insights, their therapeutic potential in humans remains uncertain, and clinical translation has not yet been established [55,56].

**Key Takeaway:** The strength of evidence supporting therapies for MIRI varies considerably. While some interventions have advanced to large randomized trials and demonstrated neutral or negative results (e.g., cyclosporine A and ischemic conditioning), others remain clinically promising but incompletely validated (e.g., metoprolol, Nicorandil, and SSO2). Most molecular and epigenetic strategies currently rely on preclinical evidence alone. Therefore, mechanistic plausibility should not be equated with clinical efficacy, and future studies should prioritize rigorous translational validation before incorporation into routine STEMI care.

## 21. Why Have Cardioprotective Strategies for MIRI Failed to Translate into Clinical Practice?

Despite more than three decades of intensive research, myocardial ischemia–reperfusion injury remains one of the most challenging therapeutic targets in cardiovascular medicine. Numerous interventions have demonstrated substantial infarct size reduction and improved cardiac function in experimental models; however, the vast majority have failed to reproduce these benefits in large clinical trials. This translational gap represents one of the defining challenges in contemporary cardioprotection research.

One of the most notable examples is cyclosporine A, an inhibitor of mitochondrial permeability transition pore (mPTP) opening. Early experimental studies and small proof-of-concept clinical trials suggested that cyclosporine could reduce reperfusion injury and limit infarct size. However, larger randomized trials, including the CIRCUS and CYCLE studies, failed to demonstrate significant improvements in clinical outcomes, left ventricular remodeling, or mortality. Similar disappointments have been observed with multiple other cardioprotective interventions targeting oxidative stress, inflammation, mitochondrial dysfunction, and calcium overload.

Likewise, ischemic conditioning strategies, including ischemic preconditioning, postconditioning, and remote ischemic conditioning, produced highly promising results in preclinical studies. Although initial clinical investigations suggested potential benefits, subsequent large multicenter trials failed to consistently demonstrate reductions in infarct size or improvements in long-term cardiovascular outcomes. These findings have raised important questions regarding the applicability of experimental cardioprotection paradigms to real-world STEMI populations.

Several factors likely contribute to these translational failures. Experimental studies are commonly performed in young, healthy animals without the complex comorbidity burden encountered in clinical practice. In contrast, patients presenting with STEMI are frequently older and often have diabetes mellitus, hypertension, obesity, chronic kidney disease, dyslipidemia, or prior cardiovascular disease. These conditions may fundamentally alter myocardial metabolism, inflammatory responses, microvascular function, and susceptibility to cardioprotective interventions [57].

Diabetes deserves particular attention because hyperglycemia, insulin resistance, endothelial dysfunction, and mitochondrial abnormalities may impair many of the signaling pathways responsible for endogenous cardioprotection, including the RISK and SAFE pathways. Similarly, aging is associated with reduced mitochondrial reserve, impaired autophagy, increased oxidative stress, and chronic low-grade inflammation, all of which may attenuate responses observed in experimental models.

Another major challenge is the increasing complexity of modern STEMI treatment. Contemporary patients routinely receive potent antiplatelet agents, statins, beta-blockers, renin–angiotensin system inhibitors, anticoagulants, and rapid reperfusion therapy. Many of these treatments possess intrinsic cardioprotective properties that may reduce the ability to detect additional benefits from novel interventions. Furthermore, interactions between investigational therapies and standard-of-care medications remain incompletely understood.

Timing also represents a critical limitation. Many experimental therapies must be administered before ischemia or immediately at reperfusion to achieve maximal efficacy. In clinical practice, however, treatment initiation is often delayed, and the precise onset of myocardial ischemia is frequently uncertain. As a result, the therapeutic window identified in animal models may not be reproducible in patients.

The selection of study endpoints further complicates translation. Experimental studies often focus on infarct size reduction under highly controlled conditions, whereas clinical trials typically evaluate composite outcomes such as mortality, recurrent myocardial infarction, heart failure hospitalization, or major adverse cardiovascular events. Because these outcomes are influenced by numerous factors beyond reperfusion injury, demonstrating clinically meaningful benefit becomes substantially more difficult [57,58].

Finally, the field has increasingly recognized a broader reproducibility crisis in cardioprotection research. Differences in animal species, experimental protocols, ischemia duration, reperfusion methods, infarct quantification techniques, and statistical approaches have contributed to inconsistent findings across laboratories. In response, international initiatives such as the CAESAR consortium have emphasized the importance of rigorous experimental design, multicenter validation, and standardized methodologies before translation into human studies.

Collectively, these observations suggest that the failure of many cardioprotective therapies does not necessarily invalidate the biological importance of MIRI. Rather, it highlights the complexity of translating mechanistic discoveries into effective clinical interventions. Future progress will likely require improved preclinical models that better reflect real-world STEMI populations, more precise patient phenotyping, integration of coronary physiology and cardiac imaging biomarkers, and personalized approaches targeting specific reperfusion injury phenotypes [59,60].

**Key Takeaway:** The history of MIRI research is characterized by a striking disconnect between robust preclinical success and limited clinical translation. Differences in patient characteristics, comorbidities, aging, concomitant therapies, treatment timing, endpoint selection, and experimental reproducibility have all contributed to the failure of many promising cardioprotective strategies. Understanding these limitations is essential for designing the next generation of translational cardioprotection trials.

## 22. Sex Differences in Myocardial Ischemia–Reperfusion Injury

Increasing evidence suggests that myocardial ischemia–reperfusion injury exhibits important sex-specific differences involving inflammatory activation, mitochondrial function, oxidative stress, and immune responses. Experimental studies have consistently demonstrated smaller infarct sizes and improved myocardial recovery in female animals compared with males, an effect largely attributed to the cardioprotective actions of estrogen and sex-dependent modulation of inflammatory signaling pathways.

Estrogen exerts multiple protective effects during ischemia–reperfusion by enhancing endothelial nitric oxide production, reducing oxidative stress, preserving mitochondrial function, and attenuating apoptosis. In addition, estrogen has been shown to modulate inflammatory responses through suppression of NF-κB signaling, reduced production of pro-inflammatory cytokines such as TNF-α and IL-1β, and regulation of macrophage polarization toward reparative phenotypes. These mechanisms may contribute to reduced inflammatory injury and improved myocardial resilience in premenopausal females.

Sex differences have also been observed in innate and adaptive immune responses. Female subjects generally exhibit stronger immune activation but may demonstrate more efficient resolution of inflammation and tissue repair following myocardial injury. Differences in neutrophil recruitment, macrophage polarization, inflammasome activation, and cytokine expression have all been reported in experimental models of MIRI. Furthermore, sex hormones influence mitochondrial bioenergetics and reactive oxygen species generation, potentially altering susceptibility to reperfusion-associated oxidative damage.

Clinical studies have yielded more complex findings. Women presenting with STEMI are generally older and have a higher burden of comorbidities, including hypertension, diabetes, and microvascular dysfunction. Consequently, although biological mechanisms may confer some intrinsic cardioprotection, female patients frequently experience delays in diagnosis and reperfusion therapy, which may offset these advantages. Emerging imaging studies suggest that sex-related differences may also exist in microvascular obstruction, myocardial edema, and adverse ventricular remodeling, although available data remain inconsistent [61].

**Key Takeaway:** Sex represents an important biological variable influencing the pathophysiology of MIRI through modulation of inflammatory responses, mitochondrial function, oxidative stress, and tissue repair mechanisms. While experimental evidence supports significant cardioprotective effects in females, clinical outcomes are influenced by multiple confounding factors including age, comorbidities, and treatment delays. Future cardioprotection trials should incorporate sex-specific analyses to better define therapeutic responses and support precision medicine approaches in STEMI care.

## 23. Discussion

Myocardial ischemia–reperfusion injury (MIRI) remains a major challenge in cardiovascular medicine despite significant advances in reperfusion therapies. Although rapid restoration of coronary blood flow is essential for limiting ischemic myocardial necrosis, reperfusion itself paradoxically initiates a complex cascade of oxidative, inflammatory, and metabolic disturbances that further aggravate cardiac injury. Excessive generation of reactive oxygen species (ROS) during reperfusion represents one of the central mechanisms underlying cardiomyocyte dysfunction and death. Mitochondria play a dual role in this process, acting both as a major source and target of oxidative stress. ROS accumulation contributes to mitochondrial permeability transition pore (mPTP) opening, calcium overload, ATP depletion, and activation of apoptotic and pyroptotic signaling pathways. At the same time, inflammatory activation through the NLRP3 inflammasome, Toll-like receptor signaling, neutrophil extracellular trap (NET) formation, and cytokine release amplifies tissue injury and promotes endothelial dysfunction and microvascular impairment. Emerging evidence also highlights the importance of macrophage polarization, immunometabolic regulation, and interconnected cell death pathways such as PANoptosis in the progression of MIRI.

Despite extensive experimental progress, translation of cardioprotective strategies into routine clinical practice remains limited. Multiple pharmacological and non-pharmacological interventions targeting oxidative stress, mitochondrial dysfunction, inflammation, and regulated cell death have demonstrated encouraging results in preclinical studies but inconsistent efficacy in clinical trials. This translational gap likely reflects the multifactorial nature of MIRI, the complexity of overlapping signaling pathways, variability in therapeutic timing, and the influence of metabolic comorbidities such as diabetes and hypercholesterolemia. Nevertheless, therapies focused on mitochondrial preservation, inflammasome inhibition, antioxidant modulation, macrophage reprogramming, and integrated suppression of multiple cell death pathways continue to represent promising directions for future research. A more comprehensive understanding of the molecular interplay between oxidative stress, inflammation, and immune dysregulation may facilitate the development of targeted multidimensional therapies capable of improving myocardial recovery and long-term cardiovascular outcomes following reperfusion.

## 24. Conclusions

Oxidative stress is a central mechanism in myocardial ischemia–reperfusion injury (MIRI), linking mitochondrial dysfunction, calcium overload, inflammation, and regulated cell death pathways. Excessive ROS production during reperfusion amplifies myocardial damage through activation of inflammatory signaling, endothelial dysfunction, and cardiomyocyte death, ultimately contributing to adverse cardiac remodeling and heart failure progression.

Although reperfusion therapy remains essential for myocardial salvage, effective treatments specifically targeting MIRI are still lacking. Future therapeutic strategies should focus on integrated approaches capable of simultaneously modulating oxidative stress, mitochondrial injury, inflammation, and cell death pathways in order to improve myocardial recovery and long-term cardiovascular outcomes.

## Figures and Tables

**Table 1:** Key Molecular Pathways Involved in NLRP3 Inflammasome Activation and Inflammatory Dysregulation in MIRI.

Pathway/Molecule	Main Mechanism in MIRI	Major Biological Effects	Potential Clinical/Therapeutic Implications
NLRP3 Inflammasome	Activation by DAMPs and PAMPs following ischemia–reperfusion injury	Activation of caspase-1, maturation of IL-1β and IL-18, amplification of inflammation	Potential therapeutic target for reducing myocardial inflammation and infarct size
ASC (Apoptosis-associated speck-like protein containing CARD)	Adapter protein linking NLRP3 to pro-caspase-1	Promotes inflammasome assembly and inflammatory signaling	ASC deficiency associated with reduced infarct size in experimental models
Caspase-1	Cleavage and activation through inflammasome signaling	Activation of IL-1β and IL-18, induction of pyroptosis	Caspase-1 inhibition may attenuate inflammatory myocardial injury
NF-kβ	Activated downstream of TLR signaling after DAMP/PAMP recognition	Upregulates NLRP3, pro-IL-1β, and pro-IL-18 expression	Central mediator of inflammatory amplification during reperfusion
TLR4 (Toll-like receptor 4)	Recognition of tissue injury signals and activation of inflammatory pathways	Activates NF-kβ and MyD88 signaling	Important upstream target in reperfusion-induced inflammation
MyD88	Intracellular adaptor protein mediating TLR signaling	Enhances cytokine production and inflammatory responses	Experimental inhibition reduced infarct size and reperfusion injury
IL-1β	Produced following caspase-1 activation	Amplifies cytokine release, leukocyte recruitment, extracellular matrix remodeling	Potential biomarker and therapeutic target in MIRI
IL-18	Activated by caspase-1 downstream of NLRP3	Promotes inflammatory signaling and immune activation	Associated with adverse myocardial remodeling
TNF-α	Released during inflammatory activation after reperfusion	Promotes apoptosis, inflammation, and endothelial dysfunction	Contributes to myocardial injury progression
TLR9	Activated by mitochondrial DNA released during oxidative stress	Stimulates NF-kβ-mediated inflammatory pathways	May contribute to sterile inflammation during MIRI
Mitochondrial DNA (mtDNA)	Released from damaged mitochondria during oxidative stress	Acts as a DAMP activating inflammatory pathways	Marker of mitochondrial injury and inflammation
RISK Pathway (PI3K–Akt and ERK1/2)	Pro-survival signaling cascade activated during reperfusion	Promotes cardiomyocyte survival and limits cell death	May interact with NLRP3 signaling and contribute to cardioprotection
Pyroptosis	Inflammatory programmed cell death mediated by inflammasome activation	Cardiomyocyte death and inflammatory amplification	Emerging target for cardioprotective interventions
Oxidative Stress/ROS	Excess ROS production during reperfusion activates inflammatory signaling	NLRP3 activation, mitochondrial dysfunction, endothelial injury	Central mechanism linking reperfusion injury and inflammation

**Table 2:** Clinical and Experimental Therapeutic Strategies Targeting MIRI, Classified According to Translational Stage and Current Evidence Status.

Therapeutic Strategy	Mechanism	Evidence Level	Current Status
Cyclosporine A	mPTP inhibition	Phase III	Negative
Ischemic conditioning	Endogenous cardioprotection	Phase III	Inconsistent/Negative
Metoprolol	Anti-inflammatory, anti-neutrophil	Phase II/III	Clinically promising
SSO2	Enhanced oxygen delivery	Clinical	Promising
Nicorandil	KATP channel activation	Clinical	Promising
Metformin	AMPK/RISK activation	Early clinical	Mixed
Tongxinluo	Anti-pyroptotic	Early clinical	Preliminary
Vitamin C/Glutathione	Antioxidant	Small clinical studies	Preliminary
HDAC inhibitors	Epigenetic modulation	Preclinical	Experimental
NLRP3 inhibitors	Anti-inflammatory	Preclinical/Early translational	Experimental
mtDNA targeting	Mitochondrial signaling	Preclinical	Experimental
Mitophagy stimulation	Mitochondrial quality control	Preclinical	Experimental

## Data Availability

The raw data supporting the conclusions of this article will be made available by the authors on request.
